# Side differences of upper quarter Y balance test performance in sub-elite young male and female handball players with different ages

**DOI:** 10.1186/s13102-021-00364-3

**Published:** 2021-11-01

**Authors:** Julian Bauer, Stefan Panzer, Thomas Muehlbauer

**Affiliations:** 1 Division of Movement and Training Sciences/Biomechanics of Sport, University of Duisburg-Essen, Gladbecker Str. 182, 45141 Essen, Germany; 2 Institute of Sport Science, Saarland University, 66123 Saarbrücken, Germany; 3 Department of Health and Kinesiology, Texas A&M University, College Station, TX USA

**Keywords:** Inter-limb differences, Asymmetry, Upper quarter mobility/stability, Athletes

## Abstract

**Background:**

Handball is characterised by repetitive passing and shooting actions mainly performed with the throwing arm. This can lead to side differences (inter-limb asymmetry) in upper quarter mobility/stability between the throwing and non-throwing arm, which could even increase with advancing age (i.e., playing experience). However, side differences in upper quarter mobility/stability is associated with an increased musculoskeletal injury risk. Therefore, we assessed side differences in upper quarter mobility/stability in young handball players at different ages using a cross-sectional study design.

**Methods:**

Upper Quarter Y Balance test performance of the throwing and non-throwing arm was assessed in 190 sub-elite young female and male handball players (13–18 years). Per arm, relative maximal reach distances (% arm length) for all three directions (i.e., medial, inferolateral, superolateral) and the composite score (CS) were calculated and used for an age × side analysis of variance. Additionally, partial eta-squared (*η*p^2^) was calculated as an effect size measure.

**Results:**

Irrespective of measure, statistically significant main effects of age (except for the composite score) and side but no statistically significant age × side interaction effects were detected. Further, limb asymmetry in the inferolateral reach direction was above the injury-related cut-off value (i.e., ≥ 7.75% arm length) in 13- and 14-year-olds but not in the older players.

**Conclusion:**

The detection of limb asymmetry above the proposed injury-related cut-off value in younger players (13- and 14-year-olds) but not in older players (15- to 18-year-olds) may be indicative for an increased injury risk for the younger age group. Thus, prevention programs should be implemented in the handball training routine, especially for the younger ones.

## Background

Handball is characterised by a mainly unilateral technique in terms of passing and shooting. This may lead to side differences between the throwing (TA) and non-throwing arm (NTA) (i.e., inter-limb asymmetry) in terms of upper quarter mobility/stability [1]. Differences in the reach directions, e.g. a more pronounced mobility in the medial reach direction of the TA may be advantageous for some playing positions, i.e., wingers who often shoot with a long arm to increase the angles from the wings. Backcourt players often have to shoot over a block, therefore, good mobility in the TA in the superolateral reach direction may be helpful for them. A recent study by Teyhen et al. [2], however, reported a higher risk of injuries when surpassing a certain cut-off score of side differences in upper quarter mobility/stability. More precisely, Teyhen et al. [2] reported subjects (mean age: 24.7 ± 5.2 years) with an inferolateral reach asymmetry of ≥ 7.75% arm length (AL) in the Upper Quarter Y Balance test (YBT-UQ) to be associated with an increased risk (i.e., risk ratio: 1.2, odds ratio: 1.4) of a future time-loss musculoskeletal injury. The handball-specific motor stimuli with unequal distributions between the TA and the NTA evoke adaptions in the biological structures enabling the athletes to adequately process the load [3]. However, these asymmetries in the shoulder may have detrimental effects on the kinetic chain possibly increasing the likelihood of injuries. In this regard, Wedderkopp et al. [4] reported a rate of 52 injuries/1000 match hours in 14–16 years old female players whereas Olsen et al. [5] reported a match injury rate of 8.3 injuries/1000 match hours in males and 10.4 injuries/1000 match hours in females (aged 15–18 years). A more recent study by Moeller et al. [6] reported, 15.1/1000 match hours in U-18 and 11.1/1000 match hours in U-16 players (679 players, 44% female).

The aforementioned asymmetries due to the side differences of handball-specific unilateral passing and shooting actions may additionally be modulated by playing experience (i.e., years of training/competition). In other words, especially older players may have experienced a higher overall game and training load, which may have lead to more pronounced side differences in these senior players contrary to players who are still eligible to play in youth leagues. Even though some studies [7–9] already assessed side differences between the upper extremities based on the YBT-UQ performance and did not report significant differences, it has to be outlined that the study participants ranged from untrained college students [9] over trained high school baseball/softball players [8] to overhead athletes like volleyball, basketball etc. [7]. Contrary to the aforementioned sports that are played with a much lighter ball than handball, assessing shoulder mobility/stability in handball players may give additional insights mainly because of the following reasons: First, handball is a sport in which shots and passes are executed mainly unilaterally with the TA. Second, the inferolateral reach direction closely resembles the last phase of the throwing movement (i.e., the follow-through phase) in all kinds of passing and shooting actions in handball players. Third, from childhood over adolescence to adulthood the proportion of handball-specific training load and unilateral actions steadily increases. Therefore, youth (i.e., ~ 13–18 years) marks an important time span, to determine at what age inter-limb asymmetry is prevalent, so that injury prevention programs can be implemented accordingly. Further, in each of the aforementioned studies [7–9] only one specific age group was included. Therefore, it seems interesting to investigate, whether these findings will be observable in handball players in general and in female and male youth players across different ages, specifically.

Thus, the goal of the present study was to assess side differences between the TA and NTA in terms of upper quarter mobility/stability in handball players of different ages (i.e., years of training/competition). We hypothesised that due to the handball-specific unilateral passing and shooting actions side differences in the YBT-UQ performance between the TA and the NTA would be present, increase across age groups, and reach a level, which is above the proposed cut-off value (i.e., inferolateral reach asymmetry of ≥ 7.75% AL) for an increased risk of sustaining a musculoskeletal injury, especially in the older players [2].

## Methods

### Participants

One-hundred ninety sub-elite young female (*n* = 80) and male (*n* = 110) handball players aged 13–18 years participated in this cross-sectional cohort study (see Table 1). All participants played in the highest or second highest league of their respective age group. Playing experience (i.e., years of training/competition) increased with age from 6.1 ± 1.8 years for the 13-year-olds to 10.2 ± 3.1 years for the 18-year-olds. The inclusion criteria were based on the training methodological framework of the German Handball Association [10]. All players were either in the *Basic training* (age range: 13–14 years), *Advanced training I* (age range: 15–16 years) or *Advanced training II* (age range: 17–18 years) which includes a systematic handball-specific training with predetermined training content. None of the participants reported a history of musculoskeletal injuries or neurological disorders.

**Table 1:** Characteristics of the handball players (*N* = 190) by age category

Characteristic	13 years (*n* = 25)	14 years (*n* = 32)	15 years (*n* = 49)	16 years (*n* = 34)	17 years (*n* = 33)	18 years (*n* = 17)
Sex, f/m	22/3	5/27	21/28	14/20	13/20	5/12
Body height, cm	166.2 ± 6.7	177.1 ± 8.0	177.2 ± 9.6	176.0 ± 8.6	177.7 ± 8.0	180.5 ± 8.2
Body mass, kg	57.9 ± 10.6	67.3 ± 11.9	69.2 ± 10.1	74.4 ± 12.2	72.3 ± 14.0	79.3 ± 12.1
BMI, kg/m^2^	20.0 ± 5.0	21.4 ± 2.7	22.0 ± 2.6	24.0 ± 3.4	23.0 ± 3.6	24.2 ± 2.2
Left arm length, cm	83.6 ± 4.1	89.4 ± 4.6	89.7 ± 5.6	89.3 ± 5.5	89.0 ± 5.2	91.1 ± 4.9
Right arm length, cm	83.8 ± 4.4	90.1 ± 4.8	90.1 ± 5.5	89.9 ± 5.0	89.3 ± 4.9	91.7 ± 4.7
Throwing arm, left/right	1/24	2/30	4/45	4/30	4/29	0/17
Playing experience, years	6.1 ± 1.8	6.3 ± 2.6	8.3 ± 2.3	8.3 ± 2.9	10.4 ± 2.9	10.2 ± 3.1

Values are mean ± standard deviation

*BMI* Body Mass Index, *f* female, *m* male

### Testing procedures

The measurements were carried out at the same time in the evening at the beginning of the week (i.e., Mondays) in the training venues of the clubs during the competitive phase. The YBT-UQ was executed in a non-fatigued state immediately following a standardised warm-up including 5 min of sub-maximal running at a moderate speed and a test-specific warm-up consisting of three sub-maximal reaches per arm and reach direction for each player. All participants received standardised verbal instructions and a visual demonstration of the testing procedure which consisted of the assessment of anthropometric variables (i.e., body mass, body height, arm length) followed by performance assessment in the YBT-UQ. The participants had no prior experience with the assessment of YBT-UQ performance.

#### Assessment of anthropometric variables

Arm length was assessed in centimetres with a tape measure from the seventh cervical spinous process to the distal tip of the middle finger while the arm was held in 90° abduction. Body mass (kg) was measured with a Seca 803 digital scale (Basel, Switzerland) without shoes. For body height assessment, subjects were asked to stand upright while looking forward and straight. The height was determined from the ground to the top of the subjects` head in centimetres with a stadiometer (Seca 217, Basel, Switzerland). All subjects were asked which position they mainly play and what their TA is.

#### Assessment of upper quarter Y balance test performance

YBT-UQ test performance was assessed with a Y Balance Test Kit (Move2Perform, Evansville, IN, USA) and noted down on adapted YBT-UQ scoring sheets. The participants were asked to assume a push-up position on the centralised platform with their feet shoulder-width apart. Out of this starting position, the participants moved the reach indicator into the medial (MD), inferolateral (IL), and superolateral (SL) directions. All reach directions had to be executed consecutively while maintaining the push-up position with the other arm on the centralised platform. Trials were invalid when the participants pushed the indicator boxes without keeping contact, did not keep up the push-up position on the base or lost contact on the floor with their feet. A 30-s rest period was granted after each trial. The first three trials had the right arm as the stance arm. The left arm was the stance arm for the last three trials. Each reach direction was noted down on the scoring sheet. The best trial was analysed by dividing the absolute maximal reach distance (cm) for each direction by the subject's AL (cm) and then multiplied by 100 (% AL). A composite score (CS) was calculated as a mean of the maximum reach distances for every direction and both arms, separately. Further, limb asymmetry for the IL reach direction was calculated as the absolute value of the normalized (% AL) reach differences between the TA and the NTA.

### Statistical analyses

Data are presented as group mean values ± standard deviations (SD). Normal distribution was examined and confirmed using Shapiro Wilk test. Subsequently, a 6 (age: 13, 14, 15, 16, 17, 18 years) × 2 (side: throwing arm, non-throwing arm) analysis of variance (ANOVA) with repeated measure on side was performed to analyse YBT-UQ performance. Post-hoc tests with Bonferroni-adjusted α were used to identify comparisons that were statistically significant. Additionally, partial eta-squared (*η*p^2^) was calculated as an effect size measure and classified as small (0.02 ≤ *η*p^2^ ≤ 0.12), medium (0.13 ≤ *η*p^2^ ≤ 0.25), and large (*η*p^2^ ≥ 0.26) [11]. Analyses were performed using Statistical Package for Social Sciences (SPSS) version 27.0 and the significance level was set at *p* < 0.05.

## Results

### Performance differences by age and side

The means, SDs, and the corresponding ANOVA outcomes for the normalized YBT-UQ performance are presented in Table 2. The ANOVA showed statistically significant main effects of age (except for the CS) and side. For the MD reach direction, the post-hoc analysis indicated that 13-year-old players (*p* = 0.039; TA: 109.2 ± 11.5% AL, NTA: 108.3 ± 8.5% AL) and 14-year-old players (*p* = 0.006; TA: 110.6 ± 9.0% AL, NTA: 109.5 ± 8.7% AL) achieved significantly better reach distances compared to 17-year-old players (TA: 97.9 ± 19.9% AL, NTA: 96.4 ± 17.5% AL). Regarding the SL reach direction, the post-hoc analysis yielded that 18-year-old players (*p* = 0.003; TA: 92.2 ± 10.7% AL; NTA: 89.0 ± 10.9% AL) achieved significantly better reach distances than the 13-year-old players (TA: 77.8 ± 11.0% AL, NTA: 78.4 ± 12.9% AL). Concerning the IL reach direction, the post-hoc analysis did not indicate performance differences between age categories. Further, the post-hoc analysis detected statistically significant side differences for the 14-year-olds (IL: *p* = 0.042; TA: 108.1 ± 11.7% AL, NTA: 104.5 ± 13.1% AL; SL: *p* = 0.003; TA: 83.5 ± 8.8% AL, NTA: 80.4 ± 9.8% AL; CS: *p* = 0.011; TA: 100.6 ± 8.2% AL, NTA: 98.0 ± 8.4% AL), the 15-year-olds (MD: *p* = 0.010; TA: 107.7 ± 9.4% AL, NTA: 105.6 ± 9.9% AL), the 16-year-olds (CS: *p* = 0.044; TA: 100.9 ± 8.9% AL, NTA: 99.2 ± 10.0% AL), and the 18-year-olds (SL: *p* = 0.032; TA: 92.2 ± 10.7% AL, NTA: 89.0 ± 10.9% AL). However, we did not observe any Age × Side interaction effect (all *p’s* > 0.05). Thus, the observed side differences were not affected by player's age (i.e., training/playing experience).

**Table 2:** Outcomes for the 6 (age: 13, 14, 15, 16, 17, 18) × 2 (side: throwing arm, non-throwing arm) analysis of variance with repeated measure on side

Measure	13 years (*n* = 25)		14 years (*n* = 32)		15 years (*n* = 49)		16 years (*n* = 34)			17 years (*n* = 33)		18 years (*n* = 17)		*p* value (*η*p^2^)	
	TA	NTA	TA	NTA	TA	NTA	TA		NTA	TA	NTA	TA	NTA	Age	Side
MD (% AL)	109.2 ± 11.5	108.3 ± 8.5	110.6 ± 9.0	109.5 ± 8.7	107.7 ± 9.4	105.6 ± 9.9	106.6 ± 18.4		105.3 ± 17.1	97.9 ± 19.9	96.4 ± 17.5	98.8 ± 23.3	97.2 ± 23.6	.002^†^ (.10)	.004* (.04)
IL (% AL)	99.1 ± 10.6	98.3 ± 14.0	108.1 ± 11.7	104.5 ± 13.1	99.3 ± 11.8	99.5 ± 13.0	109.1 ± 17.9		107.2 ± 18.2	105.9 ± 19.3	104.9 ± 19.7	108.9 ± 17.3	107.4 ± 16.4	.025^†^ (.07)	.034* (.02)
SL (% AL)	77.8 ± 11.0	78.4 ± 12.9	83.5 ± 8.8	80.4 ± 9.8	84.7 ± 11.6	83.2 ± 11.6	87.0 ± 10.9		85.2 ± 11.4	85.1 ± 12.5	83.7 ± 12.8	92.2 ± 10.7	89.0 ± 10.9	.006^†^ (.09)	.010* (.04)
CS (% AL)	95.4 ± 8.8	95.0 ± 10.0	100.6 ± 8.2	98.0 ± 8.4	97.2 ± 8.4	96.1 ± 9.4	100.9 ± 8.9		99.2 ± 10.0	96.3 ± 8.5	95.0 ± 8.4	100.0 ± 7.1	97.9 ± 8.0	.112 (.05)	< .001* (.07)

Values are mean ± standard deviation. Figures in brackets are effect sizes (*η*p^2^) with .02 ≤ *η*p^2^ ≤ .12 indicating small, .13 ≤ *η*p^2^ ≤ .25 indicating medium, and *η*p^2^ ≥ .26 indicating large effects

*AL* arm length, *CS* composite score, *Il* inferolateral, *MD* medial, *NTA* non-throwing arm, *SL* superolateral, *TA* throwing arm

^†^Significant age difference

*Significant side difference

### Limb asymmetry values by age

**Fig. 1:**
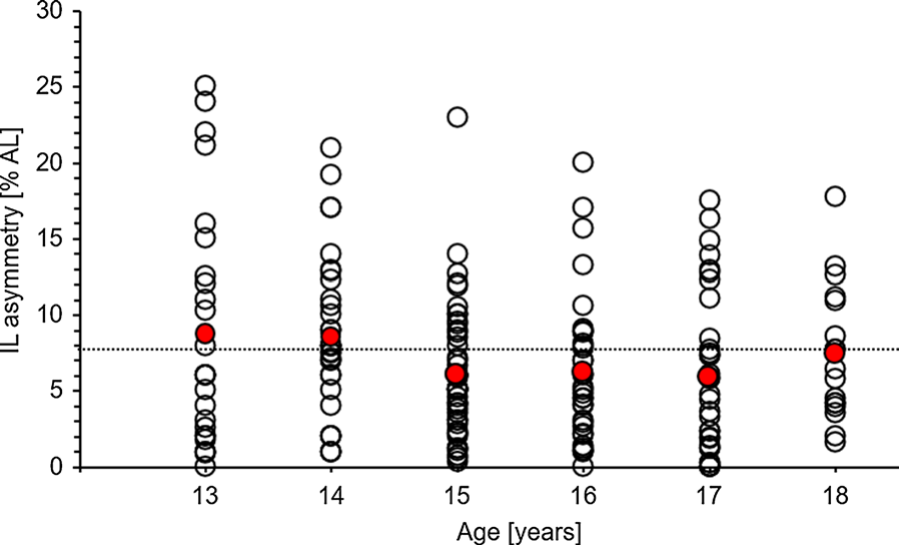
Asymmetry (% AL) for the inferolateral reach direction between the throwing and non-throwing arm by age group. The dotted line corresponds to the cut-off value of ≥ 7.75% AL that is related to an increased risk of sustaining a time-loss musculoskeletal injury [2]. Unfilled white circles represent single player values and filled red circles depict group mean values. *AL* arm length, *IL* inferolateral

Figure 1 shows the asymmetry values for the IL reach direction per player and age group. The dotted line indicates the cut-off value of ≥ 7.75% AL that is related to an increased risk of a future time-loss musculoskeletal injury [2]. On an individual level (i.e., unfilled white circles), we observed that several players obtained values above the proposed cut-off value, irrespective of age group. On a group level (i.e., filled red circles), IL reach asymmetry was above the threshold in 13-year-old players (8.72% AL) and 14-year-old players (8.57% AL) but not in the older age groups (15-year-olds: 6.07% AL, 16-year-olds: 6.26% AL, 17-year-olds: 5.94% AL, 18-year-olds: 7.39% AL).

## Discussion

The current study investigated side differences and limb asymmetry in upper quarter mobility/stability assessed via YBT-UQ in sub-elite young male and female handball players (*N* = 190) at different ages (i.e., playing experience). More specifically, we compared YBT-UQ performance between the TA and NTA in players aged 13–18 years and calculated side differences and reach asymmetry values. Our results showed: (a) significant side differences for the 14- (IL, SL, CS), the 15- (MD), the 16- (CS), and the 18-year-olds (SL), (b) IL reach asymmetry above the proposed YBT-UQ cut-off value of ≥ 7.75% AL [2] for the 13- and 14-year-olds only, and (c) YBT-UQ performance differences between ages (i.e., MD: 13- and 14-year-olds better than 17-year-olds; SL: 13-year-olds worse than 18-year-olds). Based on the age-specific normative values provided by Schwiertz et al. [12] which are based on subjects randomly chosen from urban public schools in the Ruhr metropolitan area exhibiting a wide range of physical activity, the sub-elite handball players in the present study scored higher irrespective of reach direction and arm considered compared to this cohort.

The finding of statistically significant side differences in the YBT-UQ across age groups (except for the 13- and 17-year-old players) is in accordance to our hypothesis but contrary to former studies [7–9] that reported no significant differences between the upper limbs. However, in the aforementioned studies no handball players were assessed which predominantly use one limb for passing, bouncing, and shooting actions. Based on our results, it seems that side differences do already develop in early ages and not just with advanced age (i.e., higher playing experience). This assumption can be supported by the results of this study as the asymmetry value on the YBT-LQ in the IL reach direction was above the proposed cut-off value of ≥ 7.75% AL [2] in the younger (i.e., 13- and 14-year-olds) but not in the older (15- to 18-year-olds) age groups. Even if the cut-off value was determined in US Army soldiers/warrior athletes between the ages of 18 and 45 years and not in young athletes, this may hint to the possibility for an increased risk of sustaining a future time-loss musculoskeletal injury. Based on our results (i.e., significant side differences already in young players and limb asymmetry above the cut-off value especially in young players), it can be concluded that coaches are advised to implement injury prediction and prevention programs in the handball training routine, especially for younger players. In this regard, there are already programs available that are effective in reducing the risk of upper extremity injuries [13]. More precisely, Andersson et al. [13] investigated the effects of an exercise programme designed to reduce the prevalence of shoulder problems in handball players (*N* = 660, age range: 21–24 years). Forty-five elite handball teams (intervention group: 22 teams, control group: 23 teams) were followed over the period of seven months. During this period, the intervention group executed the *Oslo Sports Trauma Research Center Shoulder Injury Prevention Programme* three times per week as a part of the handball warm-up while the control group performed regular handball training sessions. Based on equation models, the intervention group displayed a 28% lower risk of shoulder problems with small reductions in training (odds ratio: 0.72, 95% confidence interval: 0.52–0.98) and a 22% lower risk of substantial shoulder problems with moderate to severe reductions in training (odds ratio: 0.78, 95% confidence interval: 0.53–1.16) compared to the control group. In another study, Sakata et al. [14] divided 16 youth baseball teams consisting of 237 players aged 9–11 years into an intervention group (8 teams) and a control group (8 teams) and followed them over twelve months. Over this period of time, participants in the intervention group performed nine items (10 min in total) consisting of five stretching exercises, two dynamic thoracic mobility exercises, and two lower extremity balance training exercises during their normal warm-up routine. After twelve months, incidence of shoulder and elbow injuries was significantly lower in the intervention group (1.7 per 1000 athlete-exposures) compared to the control group (3.1 per 1000 athlete-exposures) (hazard ratio: 1.940, 95% confidence interval: 1.175–3.205).

Additionally, to the side differences, we also detected significant age differences in terms of YBT-UQ performance, although these were unequivocal. More precisely, for the MD reach direction younger players (i.e., 13- and 14-year-olds) performed better than older players (i.e., 17-year-olds) but for the SL reach direction younger players (i.e., 13-year-olds) performed worse than older players (i.e., 18-year-olds). Up to now, age differences in terms of YBT-UQ performance were only assessed in adults [15, 16] but not during adolescence. Therefore, only assumptions can be made on the causes of these differences. One explanation could be growth, developmental, and maturation processes. These do not occur linear but curvilinear [17] with increasing age. Growth rate, maturity status, and maturity tempo varies highly between adolescent athletes [18]. The players of the present sample may additionally represent early developers having finished the age at peak height velocity (PHV), as in youth sports better performances are often present in athletes with advanced maturity. Diverging PHV between the adolescents may therefore lead to increased tensile forces on vulnerable muscle attachments, decreased neuromuscular control, and reduced flexibility [18] which likely will have an influence on mobility and stability of the extremities, and the upper extremities being responsible for the YBT-UQ, specifically. As a consequence, large performance alterations might result within a certain duration (e.g., 1 year) [19], which for example might be more pronounced in 13-year-old players for the MD direction in contrast to the SL direction. Younger players (i.e., 13- and 14-year-olds) might have performed better in the MD direction than the older players (i.e., 17-year-olds) as the rotational capacities as well as the functional and structural adaptations could have decreased in the older players due to the higher overall training load. However, the better performances of older players in the SL direction may be present due to the training methodological framework of the German Handball Association [10]. The SL direction resembles the shooting movement, when shooting over a defensive block which is needed in older age categories (i.e., 15–18 years) as only during these age categories, it is allowed to play defensively, i.e. making it necessary to shoot over a block often positioned around the 6-m space. Contrary to this, younger players have to play more offensively, making the number of shots over a defensive block not necessary with one-on-one actions being more pronounced that are finished with all kinds of shooting techniques, i.e. variable arm positions in the younger age categories. Additionally, the training content during the so-called Basic motor education (age range: 6–12 years) focuses on a variable motor skills education during which specialised handball training and therefore numerous shoots and passes are not the main focus [10]. Consequently, the age span of 13–14 years (Basic training) is the first one with a highly handball-specific load leading to strong adaptations in terms of limb asymmetries possibly due to the higher adaptive reserves and new asymmetric stimuli especially for the TA arm at the beginning of the handball-specific career of the players. In the following after having adapted to the initial load, the handball-specific training is slightly reduced during the Advanced training I (age range: 15–16 years) and the Advanced training II (age range: 17–18 years) and athletics/motor training with higher loads becomes more pronounced (30% of the recommended training load). This more specific and higher loads athletic training (also with additional weights on a symmetrical basis) may be responsible for reducing or even neutralising the effect of the unilateral training in adolescent handball players.

The side differences were heterogeneous in terms of their extent across age groups and reach directions. However, an important underlying pattern seems to be that for example in the youngest age group (13–14 year-olds) and especially the 14-year-olds, the NTA as the stance arm (i.e., the TA as the mobile arm being tested) scores considerably worse (104.5 ± 13.1% AL) than the TA as the stance arm (108.1 ± 11.7% AL) with the NTA as the mobile arm. This pattern of the TA testing better as the stance arm and the NTA testing better as the mobile arm is present in any reach direction (Table 2) and present in 22 of the 24 combinations (age: 13, 14, 15, 16, 17, 18 years x IL, SL, MD, CS) possibly due to the NTA being responsible for a contralateral stabilising function and the TA having more mobility requirements during the varieties of passing and shooting actions.

We acknowledge that this study has some limitations that require discussion. Our findings are specific to the YBT-UQ, which is a widely used field-test for the assessment of upper quarter mobility/stability. Therefore, further research is needed to confirm our results in other types of upper-extremity functional performance tests (e.g., closed kinetic chain upper extremity stability test, seated medicine ball throw test, etc.). Additionally, the participants included in this study were sub-elite young male and female handball players who were not differentiated in terms of sexes. As an additional limitation, it needs to be stated that we based our findings on limb asymmetry alone. The future provision of specific normative values for the YBT-UQ in young female and male handball players will be helpful because a surpassing of these cut-off scores would additionally strengthen the need for specific interventions to address these deficits. Further, female and male players were unequally distributed across the six age groups. Therefore, the transfer of our findings onto equally distributed player groups should be avoided and underline the need for further investigations. Finally, care is needed when generalising the present findings to other populations of overhead athletes (e.g., basketball and/or volleyball players).

## Conclusions

Based on the detected side differences and limb asymmetry between the TA and the NTA in upper quarter mobility/stability in sub-elite young female and male handball players at different ages (13–18 years), prevention programs should be part of the handball training routine, particularly for the younger players (13–14 years) as this is the first age category with a pronounced handball-specific training load. These shoulder mobility/stability programs should include exercises to increase glenohumeral internal rotation, external rotation strength and scapular muscle strength, as well as improve the kinetic chain and thoracic mobility, preferably as a warm-up during the training [13].

## Data Availability

The data generated and analysed during the present study are not publicly available due to ethical restrictions but are available from the corresponding author upon reasonable request.

## Abbreviations

AL: Arm length
ANOVA: Analysis of variance
BMI: Body mass index
CS: Composite score
IL: Inferolateral
MD: Medial
NTA: Non-throwing arm
SD: Standard deviation
SL: Superolateral
TA: Throwing arm
\YBT-UQ: Upper Quarter Y Balance Test
